# Aberrant Promoter Methylation of YAP Gene and its Subsequent Downregulation in Indian Breast Cancer Patients

**DOI:** 10.1186/s12885-018-4627-8

**Published:** 2018-07-03

**Authors:** Sumayya Abdul Sattar Real, Farah Parveen, Asad Ur Rehman, Mohammad Aasif Khan, Sankaravamasam Venkata Suryanarayan Deo, Nootan Kumar Shukla, Syed Akhtar Husain

**Affiliations:** 1 Department of Biosciences, Jamia Millia Islamia, New Delhi, 110025 India; 2 Department of Surgical Oncology, All India Institute of Medical Science, New Delhi, 110608 India

**Keywords:** Downregulation, Hippo pathway, mRNA, Tumor suppressor gene, YAP

## Abstract

**Background:**

YAP, a potent oncogene and major downstream effector of the mammalian Hippo tumor suppressor pathway can act as either oncogene or tumor suppressor gene based on the type of tissue involved. Despite various studies, the role and mechanism through which YAP mediates its tumor suppressor or oncogenic effects are not yet fully understood. Therefore in the present study we aimed to investigate YAP at DNA, mRNA and protein level and also attempted to correlate our molecular findings with various clinicopathological variables of the patients.

**Methods:**

The study comprised of a total 137 genetically unrelated women with sporadic breast cancer cases and normal adjacent tissues not infiltrated with tumor. Mutation of YAP gene was analyzed by automated DNA sequencing. YAP promoter methylation was studied using MS-PCR. Expression at mRNA and protein level was studied using qPCR and IHC respectively.

**Results:**

In our study YAP mRNA expression was found to be 8.65 ± 6.17 fold downregulated in 67.15% cases. The expression of YAP when analyzed at the protein level by IHC was found to be absent in 78.83% cases. Results from MS-PCR analysis showed that YAP promoter methylation plays an important role in declining the expression of YAP protein. The absence of YAP protein coincided with 86.60% methylated cases thereby showing a very strong correlation (*p* = 0.001). We also investigated YAP mutation at the major check point sites in the Hippo pathway and observed no mutation. A significant association was observed on correlating mRNA expression with clinical stages (*p* = 0.038) and protein expression with ER status (*p* = 0.018) among Indian breast cancer patients.

**Conclusion:**

The expression of YAP was found to be downregulated in response to aberrant promoter methylation. The downregulation of YAP are consistent with previous studies suggesting it to have a tumor suppressive role in breast cancer. We did not observe any mutation at the major check point sites in the Hippo pathway.

**Electronic supplementary material:**

The online version of this article (10.1186/s12885-018-4627-8) contains supplementary material, which is available to authorized users.

## Background

Breast cancer accounts for 25% all cancers and is the second most common cancer in the world and the fifth cause of overall cancer mortality. Breast cancer is the most common cancer in women with 883,000 cases in less developed regions and 794,000 cases in more developed regions [1]. Breast cancer involves the interconnection of various signaling pathways [2]. Hippo signaling, an emerging tumor suppressor pathway plays a pivotal role in the development of mammary gland and breast cancer [3, 4].

YAP (Yes-associated protein) is a potent oncogene present at 11q22 amplicon and major downstream effector of the mammalian Hippo tumor suppressor pathway [5, 6]. YAP elevates invasion, proliferation, conceal apoptosis, and is adequate for transformation [7]. Cell-to-cell contacts lead to the activation of Hippo pathway which in turn leads to the phosphorylation of YAP at various serine residues including serine 127 by concerted action of LATS and MST, two uptream kinases and is secluded from the nucleus by 14-3-3 proteins thus decreasing the transcriptional activities of the target genes [6, 8]. Overexpression of YAP or its nuclear localization is frequently associated with many human cancers [9]. The tumor suppressor role of YAP is demonstrated in several studies showing its reduced level of expression in human breast cancer [10]. However, in breast cancer it is disputable of YAP being an oncogene or a tumor suppressor gene [11]. YAP can act as either oncogene or tumor suppressor gene based on the type of tissue involved [12].

Long-term existence of cancer cells requires the deregulation of diverse molecular processes [13]. Various genetic and epigenetic events in a single cell collaborated with clonal expansion and selection drives the initiation of breast cancer following its tumor progression. These events disrupt the function of gene in cancer [14, 15].

Despite various studies, the role and mechanism through which YAP mediates its tumor suppressor or oncogenic effects are not yet fully understood. To the best of our knowledge the status of YAP in Indian breast cancer patients has not been explored. In this manuscript, we have tried to investigate YAP at DNA, mRNA and protein level. We have also attempted to correlate our molecular findings with various clinicopathological variables of the patients.

## Methods

### Ethics statement

The study was approved by the Institutional Ethical Committee of All India Institute of Medical Sciences (AIIMS), New Delhi and the Institutional Human Ethical Committee of Jamia Millia Islamia, New Delhi. Written informed consent was obtained from all the participants in the study.

### Biological specimen collection

A total of 137 genetically unrelated women with sporadic breast cancer cases were included in the study. Normal adjacent breast tissue not infiltrated with tumor served as control. Inclusion criteria included female breast cancer patients in the age group 20 to 79 years with life expectancy of at least 6 months, histopathological confirmation with primary breast cancer and patients ready to consent and abide by the trial related procedures. Exclusion criteria included in the study were previous exposure to chemotherapy or radiotherapy, patients with multiple cancers or undergoing surgery for the second time and patients with acute myocardial or surgical complications. All the breast cancer cases were recruited from the Department of Surgical Oncology, AIIMS. Various clinicopathological parameters of the patients were collected in detail from their medical records (Table 1).

**Table 1:** Characteristics of study subjects (*N* = 137)

Characteristic	Cases (%)
Age (years)	
≤50	78 (56.93)
>50	59 (43.07)
Age at menarche	
≤12	26 (18.98)
>12	111 (81.02)
Menopausal status	
Premenopausal	40 (29.20)
Postmenopausal	97 (70.80)
Age at menopause	
≤45	33 (34.02)
>45	64 (65.98)
ER status	
Positive	81 (59.12)
Negative	56 (40.88)
PR status	
Positive	47 (34.31)
Negative	90 (65.69)
Her2 status	
Positive	66 (48.18)
Negative	71 (51.82)
Molecular subtypes of breast cancer	
Luminal A	46 (33.58)
Luminal B	38 (27.74)
Her2-enriched	28 (20.44)
TNBC	25 (18.25)
Tumor size	
≤5	55 (40.15)
>5	82 (59.85)
Lymph node status	
Positive	99 (72.26)
Negative	38 (27.74)
Clinical stage	
I+II	45(32.85)
III+IV	92(67.15)
Histological grade	
I+II	95 (69.34)
III	42 (30.66)

### Genomic DNA extraction

Genomic DNA was extracted from breast tumor and adjacent normal breast tissue based on the standard phenol-chlorofrom extraction method [16]. The quality and quantity of the isolated DNA was assessed by Nanodrop ND 1000 spectrophotometer (Eppendorf, Germany) and further confirmed by gel electrophoresis running on 1% agarose (Sigma-Aldrich, US) at 100mA/volt and stained with 0.5 μg/ml of ethidium bromide. The quality and quantity checkups of extracted DNA are shown in Additional file 1: Table S1. The ratio of absorbance at 260 nm and 280 nm (A_260_/A_280_) was taken to assess the purity of the DNA. ~1.8 ratio is accepted pure for DNA.

### Automated DNA sequencing

Exon 1, 2, 8 and 9 of YAP gene harbouring codons for serine 61, 109, 127, 164, 397 and lysine 494 was amplified using the following set of primers (Table 2). The PCR products underwent purification and direct sequencing carried out at Scigenome labs, Cochin using both forward and reverse pair of primers. The sequencing was repeated in order to avoid any contamination or PCR artifacts and to stringently confirm the mutation.

**Table 2:** Details of primers used in the present study

Primer	Primer Sequence	PCR product size (base pair)	Annealing Temperatu-re (°C)
Mutation primers			
YAP1 exon 1 (serine 61)	F 5’-AGGCAGAAGCCATGGATC-3’	338	56.6
	R 5’-GGTTACCTGTCGGGAGTG-3’		
YAP1 exon 2 (serine 109 and 127)	F 5’-GGCTGCAATTAAGCGCTGAC-3’	292	61.5
	R 5’-TGCTGGCAGAGGTACATCATC-3’		
YAP1 exon 2 (serine 164)	F 5’-CGAGCTCATTCCTCTCCAGC-3’	236	55.5
	R 5’-AGATAACTGTCTCCCACC-3’		
YAP1 exon 8 (serine 397)	F 5’-TTCAGACATTGCAGGACAGG-3’	248	58.8
	R 5’-CCTGTATCCATCTCATCCACAC-3’		
YAP1 exon 9 (lysine 494)	F 5’-CTCTGTGTGTTTCCACTAGG-3’	317	57.5
	R 5’-CCGGTGCATGTGTCTCCTTAG-3’		
Methylation primers			
YAP1 methylation	F 5’-AGTTCGTATAGGCGTTTCGTTC-3’	187	57.9
	F 5’-CTTAACTACAAAAAATTCTTCCGCT-3’		
YAP1 unmethylation	F 5’-AAGTTTGTATAGGTGTTTTGTTTGG-3’	188	57.9
	F 5’-CTTAACTACAAAAAATTCTTCCACT-3’		
Expression primers			
YAP1	F 5’-AAGCTGCCCGACTCCTTCTTCAAG-3’	161	64.1
	R 5’-GTCAGTGTCCCAGGAGAAACA-3’		
GAPDH	F 5’-CACTGCCACCCAGAAGACTG-3’	150	61
	R 5’-ATGCCAGTGAGCTTCCCGTT-3’		

### The Cancer Genome Atlas (TCGA)

The TCGA project (http://cancergenome.nih.gov/) constitutes genomic data analysis reservoir that has lead to the mapping of alterations in the genome in more than 11,000 human tumors across 33 types of cancer [17–19]. cBioPortal for Cancer Genomics was used to obtain the data (http://www.cbioportal.org/) [20, 21].

### Catalogue of Somatic Mutations in Cancer (COSMIC) Analysis for YAP mutations

The Catalog of Somatic Mutations in Cancer (COSMIC) database (https://cancer.sanger.ac.uk/cosmic), the largest and most comprehensive asset worldwide used to explore the influence of somatic mutations in human cancer, was executed to analyse the mutations of YAP. Pie charts were generated for overview of distribution and substitutions on the coding strand in breast cancer.

### Methylation-specific polymerase chain reaction (MS-PCR)

**Fig. 1:**
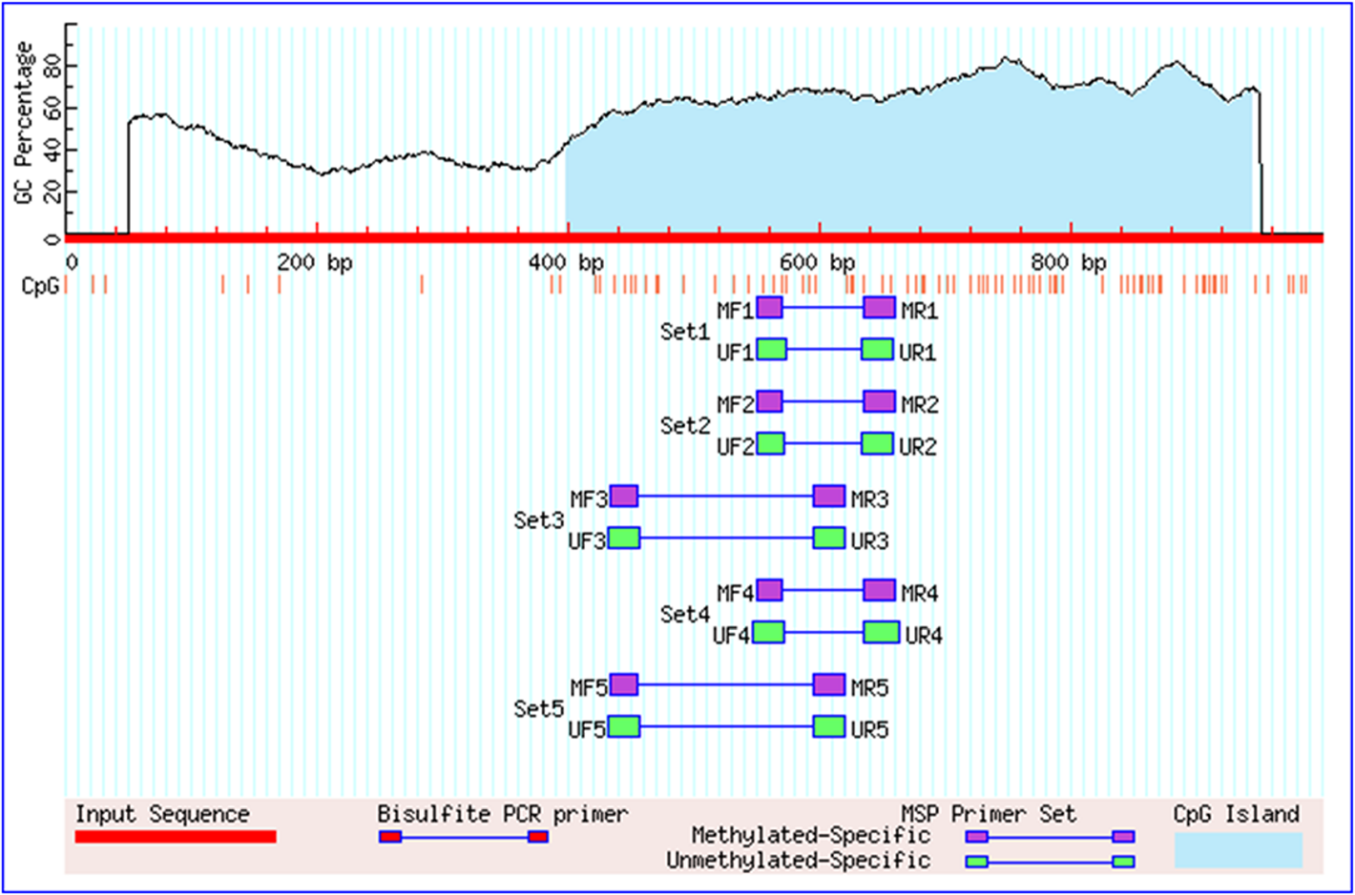
Graphical representation of CpG islands in the YAP promoter region taken from MethPrimer. Criteria used: Island size > 100, GC Percent > 50.0, Obs/Exp > 0.60

Bisulfite conversion of isolated genomic DNA was done using EZ DNA Methylation-Gold Kit (Zymo Research, Orange, CA, USA) according to the instructions given by the manufacturer. Two different sets of unmethylated and methylated YAP primers were used to amplify the bisulfite-converted product (Table 2). MethPrimer tool was used to design the primers [22]. One CpG island of 546 bp was found in the YAP promoter region when searched by Methprimer (Fig. 1). Commercially available completely unmethylated and methylated human genomic DNA (Zymo Research, Orange, CA, USA) served respectively for unmethylation and methylation positive control. Nuclease-free water instead of bisulfite-converted DNA served as negative control. 25 μl reaction volume PCR amplification was performed containing 100 ng of bisulfite-converted DNA, 1.5 mM MgCl2, 200 μM of each deoxynucleotide triphosphates (dNTPs: dATP, dCTP, dGTP, and dTTP), 0.5 μM of each forward and reverse oligonucleotide primers, 1 x PCR buffer, and 1 unit of Hot Start Taq DNA Polymerase (Qiagen, Hilden, Germany). PCR reaction was performed under following conditions : initial denaturation at 95 °C for 10 min followed by 35 cycles with denaturation at 95 °C for 45 sec, annealing at 57.9 °C for 30 sec, and extension at 72 °C for 45 sec, followed by a final extension at 72 °C for 7 min. 2% agarose gel (Sigma-Aldrich, US) containing 0.5 μg/ml of ethidium bromide was run at 100mA/volt and the PCR products were visualized, analyzed and photographed under ultraviolet (UV) illumination using Gel Doc (Bio-Rad Laboratories, CA, USA). All the experiments were repeated as an internal quality control and no distortion in the result was observed.

### Real-time polymerase chain reaction

RNA was isolated from the breast tumor and adjacent normal breast tissue stored in the RNA later (Qiagen, Hilden, Germany) by TRIzol Reagent (Invitrogen, CA, USA) according to the instructions given by the manufacturer. Later, the complementary DNA (cDNA) was synthesized using verso cDNA synthesis kit (Thermo Scientific, USA) according to the manufacturer’s instruction and was stored at -20 °C. The quantitative polymerase chain reaction (qPCR) was carried out with LightCycler® 96 SYBR Green I Master (Roche Diagnostics India Pvt. Ltd.) using the following set of primers (Table 2). GAPDH mRNA was used as an internal control, amplified in the same PCR reactions using the following primers (Table 2). PCR amplification were accordingly done : initial denaturation at 95 °C for 1 min, followed by 35 cycles with denaturation at 94 °C for 20 sec, annealing at 64.1 °C for 15 sec, and extension at 72 °C for 20 sec, followed by a final extension at 72 °C for 7 min. Quantification were performed in duplicates. Delta delta Ct method was applied to determine the relative gene expression using qPCR. LightCycler 96 Software 1.5 was used to calculate the relative amount of mRNA as the calibrator normalized ratio which was measured using the formula: RQ = 2^-∆∆Ct^, ^∆∆Ct^ = (^Ct^targeted gene – ^Ct^GAPDH) targeted sample - (^Ct^targeted gene – ^Ct^GAPDH) calibration sample. The Ct values for YAP and GAPDH mRNA are shown in Additional file 2: Table S2.

### Immunohistochemistry

Formalin-fixed tissue blocks of breast cancer samples were made which were later sectioned and obtained on poly-L-lysine coated slides. Slides were subjected to deparaffinization through various grades of xylene and rehydrated with ethanol. 0.3 % H_2_O_2_ was used to quench the internal peroxidase activity and antigen retrieval was done by boiling citrate buffer (10 mM; pH 6.0). Serum solution was used as a blocking agent to prevent non-specific interaction, and then the slides were incubated with primary antibody. YAP expression was detected by anti-YAP Mouse monoclonal Antibody (Abcam, UK). Later on, incubation with biotinylated secondary antibody against mouse and streptavidin HRP was performed for 20-30 min. 3, 3’ – diaminobenzidine (DAB) was then added to visualize the antibody binding site followed by counterstaining with hematoxylin. Normal breast tissue served as positive control and sections omitted with primary antibody served as negative control. Staining was evaluated and interpreted by expert histopathologist at 400X magnification under light microscope and graded as: (1) 0% tumor staining – no expression (2) 1% - 10% tumor staining – mild expression (3) 10% - 50% tumor staining – moderate expression (4) >50% tumor staining – high expression.

### Statistical analysis

All the statistical analysis was performed using Statistical Package of Social Science (SPSS, USA) version 17.0 for windows. The data here have been expressed as mean ± standard deviation (SD). All the comparisons between methylation status, and protein expression with the clinicopathological parameters were performed using Fisher’s exact test (two-sided). Wilcoxon signed-ranked test, a non-parametric test was applied to evaluate the significance of differences in mRNA expression levels of YAP/GAPDH mRNA. All the comparison between mRNA expression and clinicopathological parameters were performed with Kruskal-Wallis test. The *p* values < 0.05 were considered to be statistically significant. Each *p* value was statistically adjusted with Bonferroni correction.

## Results

### Downregulated YAP mRNA expression in breast cancer tissue

**Fig. 2:**
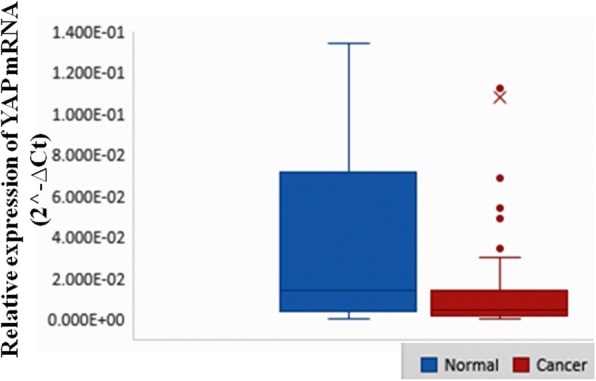
Box-and-Whisker plots showing relative expression of YAP mRNA in breast cancer and adjacent normal breast tissues. The expression of YAP mRNA in breast cancer cases were significantly lower than normal cases (*p* = 0.0001). The Y-axis represents 2^-∆Ct values for normal and cancer cases. The thick horizontal line in the box indicates the median value (1.389E-02 for normal and 4.518E-03 for cancer), the top and the bottom of the box show the 75^th^ and 25^th^ percentile values and the vertical lines extending from the box represent the largest and smallest values. Mean for normal is 2.268E0 and cancer is 1.076E-1 while Standard deviation for normal is 1.653E1 and cancer is 5.597E-1

YAP mRNA expression was detected at the mRNA level in normal and breast cancer tissues. The expression was normalized against GAPDH expression. YAP mRNA expression was found to be downregulated in 67.15% cases (92/137), out of which 60.87% cases (56/92) belonged to advanced stages III and IV of breast cancer. The 92 cases that showed downregulation were found to be 8.65 ± 6.17 fold downregulated, and the expression at mRNA level of YAP in tumor tissue was 0.11 ± 5.60 and in normal tissue was 2.27 ± 1.65 (*p* = 0.0001). The mRNA expression when correlated with different clinicopathological parameters of all the patients showed significant association with clinical stage (*p* = 0.038) (Fig. 2 and Table 3). On further analyzing the YAP mRNA expression among different molecular subtype of breast cancer cases, the highest percent downregulation was found in Her-2 enriched (78.57%) followed by TNBC (76%), Luminal B (63.16%), and Luminal A (58.70%).

**Table 3:** Correlation analysis of YAP1 mRNA expression levels with the clinical parameters in Indian breast cancer patients

Characteristic	Total (*N*)	YAP1 mRNA expression relative to GAPDH	*p* value
Normal	137	2.27 ± 1.65	0.0001
Tumor	137	0.11 ± 5.60	
Age (years)			
≤50	78 (56.93)	0.149 ± 0.00	0.464
>50	59 (43.07)	0.053 ± 0.00	
Age at menarche			
≤12	26 (18.98)	0.033 ± 0.00	0.524
>12	111 (81.02)	0.125 ± 0.00	
Menopausal status			
Premenopausal	40 (29.20)	0.036 ± 0.00	0.462
Postmenopausal	97 (70.80)	0.137 ± 0.00	
Age at menopause			
≤45	33 (34.02)	0.297 ± 1.09	0.281
>45	64 (65.98)	0.055 ± 1.98	
ER status			
Positive	81 (59.12)	0.121 ± 0.00	0.373
Negative	56 (40.88)	0.088 ± 0.00	
PR status			
Positive	47 (34.31)	0.141 ± 0.00	0.741
Negative	90 (65.69)	0.090 ± 0.00	
Her2 status			
Positive	66 (48.18)	0.053 ± 0.00	0.506
Negative	71 (51.82)	0.159 ± 0.00	
Molecular subtypes of breast cancer			
Luminal A	46 (33.58)	0.149 ± 0.00	0.731
Luminal B	38 (27.74)	0.079 ± 0.00	
Her2-enriched	28 (20.44)	0.018 ± 0.00	
TNBC	25 (18.25)	0.178 ± 0.00	
Tumor size			
≤5	55 (40.15)	0.183 ± 0.00	0.169
>5	82 (59.85)	0.057 ± 0.00	
Lymph node status			
Positive	99 (72.26)	0.109 ± 0.00	0.203
Negative	38 (27.74)	0.103 ± 0.00	
Clinical stage			
I+II	45(32.85)	0.206 ± 0.00	0.038
III+IV	92(67.15)	0.06 ± 0.00	
Histological grade			
I+II	95 (69.34)	0.096 ± 0.00	0.869
III	42 (30.66)	0.135 ± 0.00	

*p* value (Wilcoxon signed-ranked test and Kruskal-Wallis test), Bonferroni significance level *p* ≤ 0.004

### YAP protein expression is frequently absent in breast cancer

**Fig. 3:**
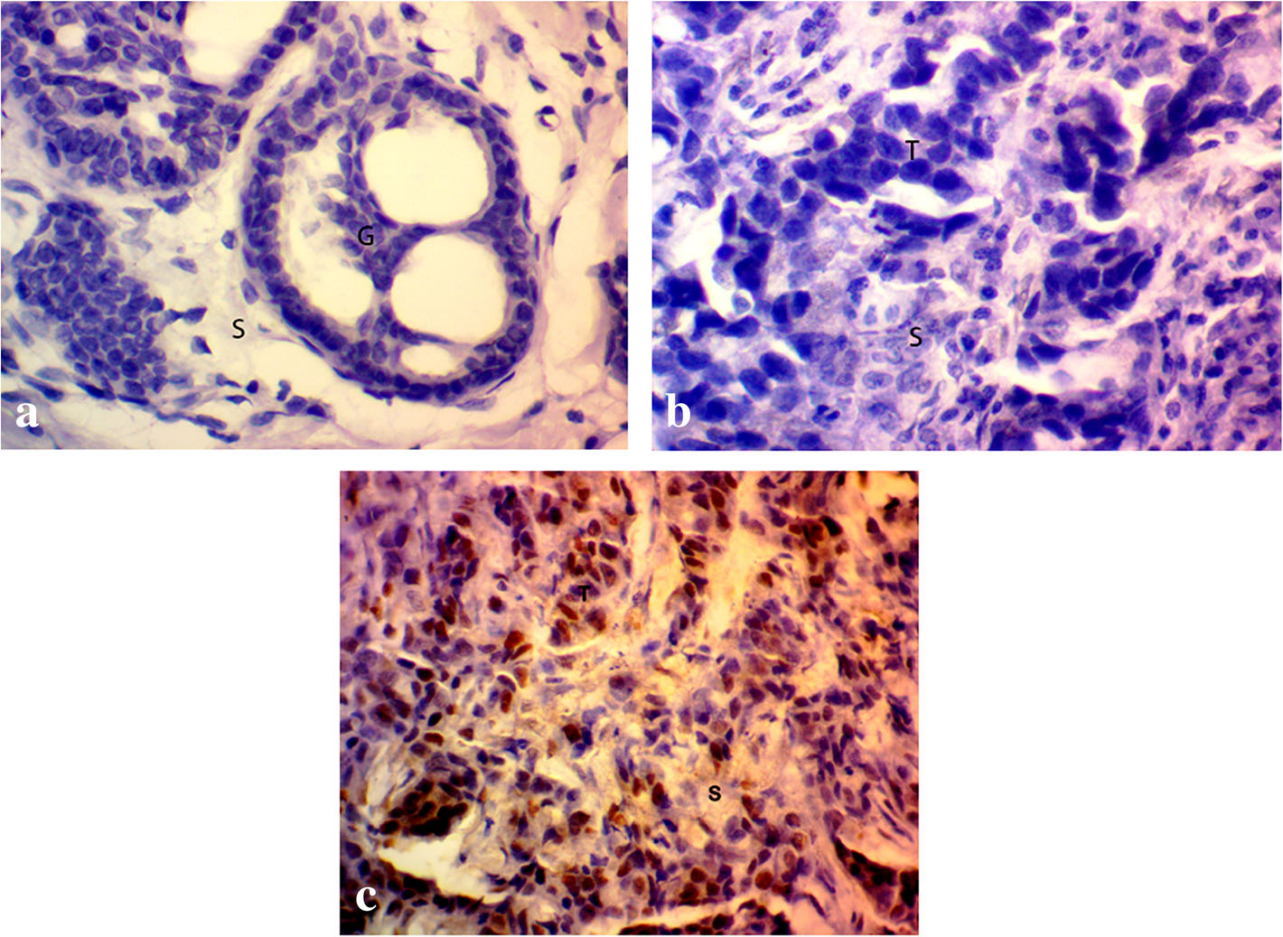
Immunohistochemical staining of human breast tissue samples by anti-YAP antibody (magnification: 400x) showing (**a**) normal breast tissue exhibiting negative YAP staining, breast tumor tissue showing (**b**) absence of YAP expression, and (**c**) moderate YAP expression. *S* stromal tissue, *G* glandular tissue, *T* tumor tissue

The expression of YAP was analyzed at the protein level by IHC and was found to be absent in 78.83% (108/137) cases. 108 cases had no or very low expression of the protein whereas remaining 29 cases (21.17%) cases had moderate to high expression of the protein (Fig. 3 and Table 5) and the percentage of YAP protein downregulation (64.81%) was higher in advanced stages III and IV of breast cancer. The percentage of YAP protein downregulation in breast cancer subtypes were different to those of YAP mRNA downregulation with 92% cases downregulation in TNBC followed by Her2-enriched (85.71%), Luminal B (73.68%), and Luminal A (71.74%) (Table 4).

**Table 4:** Correlation analysis of YAP1 protein expression levels with the clinical parameters in Indian breast cancer patients

Characteristic	Total (*N*)	YAP1 absent	YAP1 present	*p* value
Age (years)				
≤50	78 (56.93)	64 (82.05)	14 (17.95)	0.3
>50	59 (43.07)	44 (74.58)	15 (25.42)	
Age at menarche				
≤12	26 (18.98)	23 (88.46)	3 (11.54)	0.258
>12	111 (81.02)	85 (76.58)	26 (23.42)	
Menopausal status				
Premenopausal	40 (29.20)	32 (80)	8 (20)	1
Postmenopausal	97 (70.80)	76 (78.35)	21 (21.65)	
Age at menopause				
≤45	33 (34.02)	28 (84.85)	5 (15.15)	0.309
>45	64 (65.98)	48 (75)	16 (25)	
ER status				
Positive	81 (59.12)	58 (71.60)	23 (28.4)	0.018
Negative	56 (40.88)	50 (89.29)	6 (10.71)	
PR status				
Positive	47 (34.31)	38 (80.85)	9 (19.15)	0.826
Negative	90 (65.69)	70 (77.78)	20 (22.22)	
Her2 status				
Positive	66 (48.18)	52 (78.79)	14 (21.21)	1
Negative	71 (51.82)	56 (78.87)	15 (21.13)	
Molecular subtypes of breast cancer				
Luminal A	46 (33.58)	33 (71.74)	13 (28.26)	0.146
Luminal B	38 (27.74)	28 (73.68)	10 (26.32)	
Her2-enriched	28 (20.44)	24 (85.71)	4 (14.29)	
TNBC	25 (18.25)	23 (92)	2 (8)	
Tumor size				
≤5	55 (40.15)	47 (85.45)	8 (14.55)	0.139
>5	82 (59.85)	61 (74.39)	21 (25.61)	
Lymph node status				
Positive	99 (72.26)	77 (77.78)	22 (22.22)	0.816
Negative	38 (27.74)	31 (81.58)	7 (18.42)	
Clinical stage				
I+II	45(32.85)	38 (84.44)	7 (15.56)	0.373
III+IV	92(67.15)	70 (76.09)	22 (23.91)	
Histological grade				
I+II	95 (69.34)	76 (80)	19 (20)	0.653
III	42 (30.66)	32 (76.19)	10 (23.81)	

*p* value (Fisher’s Exact Test), Bonferroni significance level *p *≤ 0.005

### Association between YAP promoter methylation and YAP protein expression in breast cancer

**Fig. 4:**
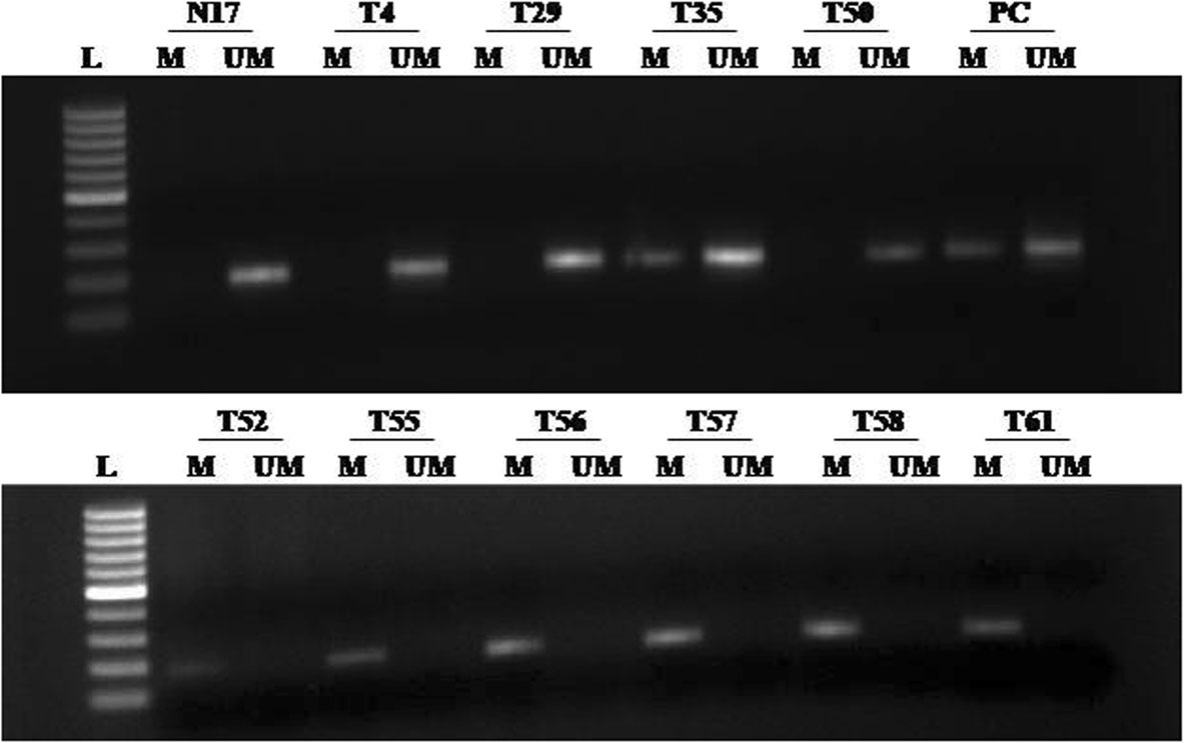
Methylation-specific PCR analysis of YAP gene in breast cancer patients: *L* 1kb DNA ladder, *M* methylated YAP promoter (PCR product size-187 bp), *UM* unmethylated YAP promoter (PCR product size-188 bp), *PC* positive control for methylated and unmethylated alleles (Completely methylated and unmethylated DNA controls, respectively), *N* normal breast sample, and *T* breast tumor sample

The methylation status of the YAP promoter was studied through methylation-specific polymerase chain reaction (MS-PCR). The results showed that YAP promoter methylation plays an important role in declining the expression of YAP protein. The absence of YAP protein coincided with 86.60% (84/97) methylated cases, whereas YAP protein was present in 13.40% (13/97) methylated cases. Only in 60% (24/40) cases where there was no methylation showed the absence of YAP protein. Further the degree of methylation was 77.78% (84/108) in cases which had downregulation of YAP protein as compared to 44.83% (13/29) cases which had moderate to high protein expression. Therefore, a very strong correlation was observed between YAP promoter methylation and YAP protein expression (*p* = 0.001) (Fig. 4 and Table 5).

**Table 5:** Correlation analysis of YAP1 promoter methylation with protein expression in Indian breast cancer patients

YAP1 protein expression	YAP Promoter				
	Methylated (% within Protein expression)	Unmethylated (% within Protein expression)	Total	*p* value	OR (95% CI)
Present	13 (44.83%)	16 (55.17%)			
Absent	84 (77.78%)	24 (22.22%)			
Total (%)	97 (70.80%)	40 (29.20%)	137	0.001	0.232 (0.098 - 0.549)
YAP Promoter	YAP1 protein expression				
	Present	Absent			
Methylated (% within methylation status)	13 (13.4%)	84 (86.6%)			
Unmethylated (% within unmethylation status)	16 (40.0%)	24 (60.0%)			
Total (%)	29 (21.17%)	108 (78.13%)	137		

*p* value *p *≤ 0.005 is considered significant

### Association between YAP promoter methylation and clinicopathological parameters in breast cancer

The promoter methylation when correlated with different clinicopathological parameters of all the patients showed no significant association. In an aggressive stage III and IV of breast cancer around 68.48% (63/92) cases were found to be methylated (Table 6).

**Table 6:** Correlation analysis of YAP1 promoter methylation with the clinical parameters in Indian breast cancer patients

Characteristics	Cases (%)	Unmethylated (%)	Methylated (%)	*p* value
Age (years)				
≤50	78 (56.93)	21 (26.92)	57 (73.08)	0.571
>50	59 (43.07)	19 (32.20)	40 (67.80)	
Age at menarche				
≤12	26 (18.98)	8 (30.77)	18 (69.23)	0.815
>12	111 (81.02)	32 (28.83)	79 (71.17)	
Menopausal status				
Premenopausal	40 (29.20)	12 (30)	28 (70)	1
Postmenopausal	97 (70.80)	28 (28.87)	69 (71.13)	
Age at menopause				
≤45	33 (34.02)	12 (36.36)	21 (63.64)	0.249
>45	64 (65.98)	16 (25)	48 (75)	
ER status				
Positive	81 (59.12)	27 (33.33)	54 (66.67)	0.252
Negative	56 (40.88)	13 (23.21)	43 (76.79)	
PR status				
Positive	47 (34.31)	12 (25.53)	35 (74.47)	0.556
Negative	90 (65.69)	28 (31.11)	62 (68.89)	
Her2 status				
Positive	66 (48.18)	18 (27.27)	48 (72.73)	0.708
Negative	71 (51.82)	22 (30.99)	49 (69.01)	
Molecular subtypes of breast cancer				
Luminal A	46 (33.58)	16 (34.78)	30 (65.22)	0.584
Luminal B	38 (27.74)	12 (31.58)	26 (68.42)	
Her2-enriched	28 (20.44)	6 (21.43)	22 (78.57)	
TNBC	25 (18.25)	6 (24)	19 (76)	
Tumor size				
≤5	55 (40.15)	15 (27.27)	40 (72.73)	0.707
>5	82 (59.85)	25 (30.49)	57 (69.51)	
Lymph node status				
Positive	99 (72.26)	29 (29.29)	70 (70.71)	1
Negative	38 (27.74)	11 (28.95)	27 (71.05)	
Clinical stage				
I+II	45(32.85)	11 (24.44)	34 (75.56)	0.43
III+IV	92(67.15)	29 (31.52)	63 (68.48)	
Histological grade				
I+II	95 (69.34)	27 (28.42)	68 (71.58)	0.839
III	42 (30.66)	13 (30.95)	29 (69.05)	

*p* value (Fisher’s Exact Test), Bonferroni significance level *p *≤ 0.005

### Association between YAP protein expression and clinicopathological parameters in breast cancer

The protein expression when correlated with different clinicopathological parameters of all the patients showed significant association with ER status (*p* = 0.018). Of the 137 cases 92 cases belonged to advanced stage III and IV of breast cancer and 76.09% (70/92) cases had absence of YAP protein (Table 4). However, 84.13% (53/63) cases of stage III and IV had no YAP protein expression and had YAP promoter methylation (Table 7).

**Table 7:** Correlation analysis of methylation and protein expression in samples having methylated YAP1 promoter or YAP1 expression loss with the clinical parameters in Indian breast cancer patients

Characteristic	Total (*N*)	Methylated YAP1		*p* value	Total (*N*)	YAP1 loss		*p* value
		YAP1 Absent	YAP1 Present			Unmethylated YAP1	Methylated YAP1	
Age (years)								
≤50	57	50	7	0.767	64	14	50	1
>50	40	34	6		44	10	34	
Age at menarche								
≤12	18	17	1	0.451	23	6	17	0.585
>12	79	67	12		85	18	67	
Menopausal status								
Premenopausal	28	23	5	0.512	32	9	23	0.447
Postmenopausal	69	61	8		76	15	61	
Age at menopause								
≤45	21	21	0	0.095	28	7	21	0.388
>45	48	40	8		48	8	40	
ER status								
Positive	54	45	9	0.375	58	13	45	1
Negative	43	39	4		50	11	39	
PR status								
Positive	35	30	5	1	38	8	30	1
Negative	62	54	8		70	16	54	
Her2 status								
Positive	48	42	6	1	52	10	42	0.497
Negative	49	42	7		56	14	42	
Molecular subtypes of breast cancer								
Luminal A	30	25	5	0.837	33	8	25	0.87
Luminal B	26	22	4		28	6	22	
Her2-enriched	22	20	2		24	4	20	
TNBC	19	17	2		23	6	17	
Tumor size								
≤5	40	36	4	0.549	47	11	36	0.819
>5	57	48	9		61	13	48	
Lymph node status								
Positive	70	61	9	0.751	77	16	61	0.613
Negative	27	23	4		31	8	23	
Clinical stage								
I+II	34	31	3	0.533	38	7	31	0.629
III+IV	63	53	10		70	17	53	
Histological grade								
I+II	68	59	9	1	76	17	59	1
III	29	25	4		32	7	25	

*p* value (Fisher’s Exact Test), Bonferroni significance level *p *≤ 0.0025

Correlation between methylation and protein expression of YAP with various clinical characteristics of Indian breast cancer patients showed that more aggressive stage III and IV of breast cancer cases had YAP protein loss significantly correlating with the aberrant YAP promoter methylation (*p* = 0.016) compared to less aggressive stage I and II of breast cancer cases (*p* = 0.05). YAP loss in methylated samples was also prevalent in cases having aggressive breast phenotype characteristics with positive lymph node status (*p* < 0.002), larger size of tumor (*p* < 0.005), and PR negative status (*p* < 0.003) (Table 8).

**Table 8:** Correlation analysis between methylation and protein expression of YAP1 in stratification by various clinical characteristics in Indian breast cancer patients

Characteristic	Total (*N*)	YAP1 methylation status	YAP1 expression		*p* value
			Absent	Present	
Age (years)					
≤50	78 (56.93)	M	50	7	0.046
		UM	14	7	
>50	59 (43.07)	M	34	6	0.017
		UM	10	9	
Age at menarche					
≤12	26 (18.98)	M	17	1	0.215
		UM	6	2	
>12	111 (81.02)	M	67	12	0.002
		UM	18	14	
Menopausal status					
Premenopausal	40 (29.20)	M	23	5	0.677
		UM	9	3	
Postmenopausal	97 (70.80)	M	61	8	<0.0007
		UM	15	13	
Age at menopause					
≤45	33 (34.02)	M	21	0	0.003
		UM	7	5	
>45	64 (65.98)	M	40	8	0.016
		UM	8	8	
ER status					
Positive	81 (59.12)	M	45	9	<0.002
		UM	13	14	
Negative	56 (40.88)	M	39	4	0.615
		UM	11	2	
PR status					
Positive	47 (34.31)	M	30	5	0.205
		UM	8	4	
Negative	90 (65.69)	M	54	8	<0.003
		UM	16	12	
Her2 status					
Positive	66 (48.18)	M	42	6	0.014
		UM	10	8	
Negative	71 (51.82)	M	42	7	0.057
		UM	14	8	
Molecular subtypes of breast cancer					
Luminal A	46 (33.58)	M	25	5	0.036
		UM	8	8	
Luminal B	38 (27.74)	M	22	4	0.045
		UM	6	6	
Her2-enriched	28 (20.44)	M	20	2	0.191
		UM	4	2	
TNBC	25 (18.25)	M	17	2	1
		UM	6	0	
Tumor size					
≤5	55 (40.15)	M	36	4	0.193
		UM	11	4	
>5	82 (59.85)	M	48	9	<0.005
		UM	13	12	
Lymph node status					
Positive	99 (72.26)	M	61	9	<0.002
		UM	16	13	
Negative	38 (27.74)	M	23	4	0.39
		UM	8	3	
Clinical stage					
I+II	45(32.85)	M	31	3	0.05
		UM	7	4	
III+IV	92(67.15)	M	53	10	0.016
		UM	17	12	
Histological grade					
I+II	95 (69.34)	M	59	9	0.02
		UM	17	10	
III	42 (30.66)	M	25	4	0.05
		UM	7	6	

*p* value (Fisher’s Exact Test), Bonferroni significance level *p *≤ 0.0025

### YAP mutation in human breast cancer

COSMIC database v72 provides over four million variants across various cancer types. COSMIC was used to generate the pie chart which had the information of mutations of substitution nonsense, missense, synonymous, insertion frame shift, and inframe deletion. 57.14% and 14.29% were respectively the substitution missense rate and substitution synonymous rate of mutant samples of breast cancer (Additional file 3: Figure S1A). YAP coding strand had 40.00% C > T and 60.0% G > A mutation in breast cancer.

### YAP TCGA database in human breast cancer

Researchers are provided with huge genome and clinical data through web portals and FTP services in TCGA breast cancer database. TCGA database on YAP gene in breast cancer makes available 108 cases affected by 102 mutations across 22 projects. The distribution of the cases is shown in Additional file 3: Figure S1B. The data demonstrates 4 somatic mutations of YAP gene in breast cancer all with low to moderate impact factor.

### YAP is not mutated at the major check point sites in the Hippo pathway

No mutation was observed in any of the codons coding for serine 61, 109, 127, 164, 397, and lysine 494.

## Discussion

The data here demonstrated the downregulation of YAP mRNA expression by 67.15%. The majority of cases (60.87%) found to be downregulated belonged to advanced stages III and IV of breast cancer and showed a significant correlation (*p* = 0.038) with clinical stage of breast cancer. At the protein level, YAP was found to be downregulated in 78.83% cases of breast cancer and these cases had either no or very low expression of YAP protein. A possible explanation for difference in YAP mRNA and protein expression can be due to varied post-transcriptional or post-translational modifications or silencing, half lives of mRNA and protein, or due to presence of significant error and noise in mRNA and protein experiments [23–25]. We also observed absence of YAP protein in normal breast tissues. It may be due to the pathological process which also affects histologically normal adjacent breast tissue apart from tumor tissue.

As consistent with YAP mRNA result the percentage of YAP protein downregulation (64.81%) was higher in advanced stages III and IV of breast cancer. The downregulation of YAP are consistent with previous studies suggesting it to have a tumor suppressive role in breast cancer [10–12, 26]. As reported earlier, in response to DNA-damage YAP mediates its tumor suppressor role by binding to p73, a family member p53 and increases p73 ability to induce apoptosis by activating apoptotic pathway [27].

Molecular subtypes of breast cancer showed different degree of YAP protein downregulation highest being TNBC followed by Her2-enriched, Luminal B, and Luminal A. This data is also consistent with earlier study indicating YAP to express differentially according to molecular subtype of cancer [5, 28]. However, we got different percent of downregulation in various subtypes of breast cancer compared to previous study [28]. On correlating the YAP protein expression with various clinicopathological parameters of Indian breast cancer cases we found a significant association with ER status (*p* = 0.018). On further analysis we found YAP to be absent for 89.29% in ER negative compared to 71.60% in ER positive. These observations are consistent with the previous study that loss of YAP is associated with ER negativity and that YAP may be a transcriptional coactivator of ER [10, 29]. While no such association was found among YAP expression and PR status as reported earlier [10]. These may be due to differential expression among diverse population.

Gene expression, genetic stability, and genomic structure may be altered by aberrant DNA methylation that can lead to carcinogenesis and tumor progression [30]. Promoter hypermethylation of critical growth regulators like tumor suppressor genes and its subsequent transcription silencing plays a pivitol role in causing cancer [31]. A recent study demonstrated hypomethylation of YAP promoter promotes the expression of YAP in polycystic ovary syndrome [32]. However, methylation status of YAP promoter in breast cancer is not yet known. Our study determines a considerable role of YAP promoter methylation in declining the expression of YAP protein. We got 70.80% (97/137) methylated breast cancer cases out of which 84 cases had absence of YAP protein thereby showing significantly high correlation (*p* = 0.001). The remaining 13 methylated cases showed the expression of YAP protein. A possible explanation to these results can be due to incomplete promoter methylation and that for methylation to silence a particular gene it should attain certain level of methylation to cross the protective boundary and once achieved the loss of transcription is stable [33]. The presence of unmethylated bands along with methylated bands in MS-PCR in tumor samples could also be explained by the above phenomena. This band may be also due to contamination of normal cells in the tumor samples. Contrary, only 60% (24/40) cases which were unmethylated for YAP promoter showed the loss of YAP protein, suggesting methylation to contribute only partially to protein loss and other molecular mechanism like genetic mutation, genomic deletions, certain transcriptional and post-transcriptional silencing, and post-translational modifications may contribute to YAP protein loss. We found no significant association when YAP promoter methylation was correlated with various clinicopathological parameters of Indian breast cancer cases.

Further analysis showed that 84.13% cases of advanced breast cancer stages III and IV had YAP promoter methylation and subsequent protein loss. Correlating methylation and protein expression of YAP with various clinicopathological characteristics of Indian breast cancer patients showed a significant association with advanced stage III and IV of breast cancer (*p* = 0.016). Majority of cases with YAP loss in methylated samples showed significance with aggressive breast cancer characteristics like lymph node status, larger size of tumor, and PR negative status (*p* < 0.005).

We evaluated the functional effect of the COSMIC mutations considering the possibility of YAP mutation to be oncogenic. Using YAP as a model gene to cross-examine the findings of COSMIC and TCGA, we report that even genes with mutations of known oncogenic functions do not necessarily contribute to growth advantage or other hallmarks of driver gene aetiology.

Studies have shown that LATS1 phosphorylates YAP at multiple sites including serine 61, 109, 127, 164, and 397 thereby inhibiting its nuclear translocation [6, 34, 35]. It was also demonstrated that mutations of all the phosphorylation sites abolishes the phosphorylation of YAP by LATS1 [28, 29]. Monomethylation of YAP at lysine 494 demonstrates a methylation dependent checkpoint in the Hippo pathway through cytoplasmic retention. Mutation at this site does not retain YAP cytoplasmically [36]. Based on all these observations we tried to check the mutation of YAP gene in exon 1, 2, 8, and 9 which had codons coding for serine 61, 109, 127, 164, 397, and lysine 494. We did not observe any mutation in the codons coding these residues. These results suggest that mutation at these sites may not be involved in the development and progression of breast cancer. However, further studies in larger sample size and in different populations are required to confirm the role of mutations at these sites.

As reported in literature, the function of YAP on being a tumor suppressor or oncogene remains controversial. In many solid tumors YAP promotes tumor growth, followed by its progression and metastasis [37]. Hippo pathway is inactivated by any changes in the tissue microenvironment or stimulation of cell by intracellular or extracellular signals thereby hyperactivating YAP. The hyperactivated YAP enters nucleus and interacts with DNA-binding transcription factors like TEA domain/Transcription Enhancer Factor (TEAD) family, p63/p73, ErbB, Smad, and RUNX1/2 [38, 39]. TEAD, a key transcription factor in hippo pathway acting downstream from YAP is required for the gene expression, cell growth, epithelial to mesenchymal transition, anchorage-independent growth, and oncogenic transformation of YAP [11, 37, 39]. YAP binds to TEAD family of transcription factors and stimulates the downstream transcription of anti-apoptotic and proliferative genes; thereby promoting oncogenesis [39]. Although several studies have demonstrated YAP to be an oncogene our data and results from other studies [10–12, 26, 38] supports its role to be tumor suppressor.

## Conclusion

To conclude, the role of YAP gene in breast cancer is still controversial. The location of YAP 11q22 is frequently associated with loss of heterozygosity (LOH) [26]. Several studies have shown decreased expression of YAP protein in breast cancer using immunohistochemistry [10, 26–28]. As revealed by LOH analysis, the loss of protein correlates with specific deletion of YAP gene locus [26]. Furthermore, YAP being coactivators of ER and PR receptors in breast cancer YAP negativity correlates with ER negativity [10]. The observations from all these studies and our results support YAP to be a tumor suppressor gene. Further large-scale study on different population is required to characterize the role of YAP in breast cancer. To the best of our knowledge we are the first to report aberrant promoter methylation of YAP and significant association with its downregulation in Indian breast cancer patients. We are also the first to report the absence of mutation at the major check point sites of YAP gene in Indian breast cancer cases.

## Additional files

Additional file 1: Quality and quantity checkups of extracted DNA. Concentration and purity of extracted genomic DNA are shown in Additional file 1: Table S1. The ratio of absorbance at 260 nm and 280 nm (A_260_/A_280_) was taken to assess the purity of the DNA. ~1.8 ratio is accepted pure for DNA. (DOCX 19 kb)

Additional file 2: Ct values of YAP and GAPDH mRNA. The Ct values for YAP and GAPDH mRNA for breast cancer cases and adjacent normal breast tissues used as control are shown in Additional file 2: Table S2. YAP mRNA expression was detected at the mRNA level in breast tumor and adjacent normal breast tissue using Real-time PCR. The expression was normalized against GAPDH expression. (DOCX 18 kb)

Additional file 3: YAP mutation in human breast cancer. TCGA and COSMIC database performed to analyse the mutations of YAP are shown in Additional file 3: Figure S1. The pie chart showing the information of mutations was generated using COSMIC databse. The data obtained using the cBioPortal for Cancer Genomics are shown in 108 cases affected by 102 mutations across 22 projects according to TCGA database. (TIF 4332 kb)

## Abbreviations

ER: Estrogen receptor
HER-2: Human epidermal growth factor receptor 2
IHC: Immunohistochemistry
PR: Progesterone receptor
TNBC: Triple-negative breast cancer
YAP: Yes-associated protein
