# Supplementary material for: Aberrant Promoter Methylation of YAP Gene and its Subsequent Downregulation in Indian Breast Cancer Patients
Source: BMC Cancer. 2018 Jul 3;18:711. doi: 10.1186/s12885-018-4627-8 (PMC6031145; doi:10.1186/s12885-018-4627-8)
Supplement: Supplementary file 1 — Quality and quantity checkups of extracted DNA. Concentration and purity of extracted genomic DNA are shown in Additional file 1: Table S1. The ratio of absorbance at 260 nm and 280 nm (A260/A280) was taken to assess the purity of the DNA. ~1.8 ratio is accepted pure for DNA. (DOCX 19 kb) [file 12885_2018_4627_MOESM1_ESM.docx]

| **Table S1. Quality and quantity checkups of extracted DNA** | | |
| --- | --- | --- |
| **Sample Number** | **Concentration of genomic DNA (ng/µl)** | **Purity of DNA (A_260_/A_280_)** |
| 1N | 500 | 1.81 |
| 1C | 550 | 1.87 |
| 2N | 625 | 1.78 |
| 2C | 700 | 1.8 |
| 3N | 450 | 1.9 |
| 3C | 600 | 1.82 |
| 4N | 514 | 1.88 |
| 4C | 545 | 1.84 |
| 5N | 777 | 1.84 |
| 5C | 780 | 1.82 |
| 6N | 657 | 1.81 |
| 6C | 1120 | 1.8 |
| 7N | 467 | 1.67 |
| 7C | 678 | 1.79 |
| 8N | 450 | 1.76 |
| 8C | 550 | 1.79 |
| 9N | 993 | 1.8 |
| 9C | 1450 | 1.82 |
| 10N | 670 | 1.85 |
| 10C | 760 | 1.84 |
| 11N | 1120 | 1.89 |
| 11C | 1450 | 1.83 |
| 12N | 650 | 1.9 |
| 12C | 670 | 1.86 |
| 13N | 750 | 1.8 |
| 13C | 825 | 1.81 |
| 14N | 590 | 1.81 |
| 14C | 610 | 1.83 |
| 15N | 1540 | 1.78 |
| 15C | 1640 | 1.77 |
| 16N | 450 | 1.81 |
| 16C | 510 | 1.82 |
| 17N | 413 | 1.89 |
| 17C | 1008 | 1.81 |
| 18N | 1130 | 1.8 |
| 18C | 1540 | 1.8 |
| 19N | 540 | 1.79 |
| 19C | 487 | 1.78 |
| 20N | 448 | 1.67 |
| 20C | 467 | 1.78 |
| 21N | 883 | 1.77 |
| 21C | 1924 | 1.81 |
| 22N | 425 | 1.84 |
| 22C | 419 | 1.85 |
| 23N | 870 | 1.8 |
| 23C | 890 | 1.81 |
| 24N | 314 | 1.87 |
| 24C | 901 | 1.83 |
| 25N | 342 | 1.71 |
| 25C | 446 | 1.77 |
| 26N | 711 | 1.79 |
| 26C | 762 | 1.8 |
| 27N | 489 | 1.81 |
| 27C | 1350 | 1.82 |
| 28N | 667 | 1.81 |
| 28C | 1737 | 1.8 |
| 29N | 774 | 1.78 |
| 29C | 1847 | 1.79 |
| 30N | 745 | 1.8 |
| 30C | 845 | 1.82 |
| 31N | 611 | 1.82 |
| 31C | 944 | 1.8 |
| 32N | 828 | 1.83 |
| 32C | 553 | 1.81 |
| 33N | 910 | 1.83 |
| 33C | 832 | 1.87 |
| 34N | 397 | 2.78 |
| 34C | 775 | 2.84 |
| 35N | 884 | 1.76 |
| 35C | 945 | 1.78 |
| 36N | 436 | 2.66 |
| 36C | 310 | 1.54 |
| 37N | 2350 | 1.83 |
| 37C | 2434 | 1.79 |
| 38N | 1339 | 1.8 |
| 38C | 1545 | 1.82 |
| 39N | 720 | 1.75 |
| 39C | 845 | 1.78 |
| 40N | 320 | 1.54 |
| 40C | 450 | 1.6 |
| 41N | 560 | 1.97 |
| 41C | 657 | 1.95 |
| 42N | 2445 | 1.79 |
| 42C | 2650 | 1.8 |
| 43N | 679 | 1.82 |
| 43C | 780 | 1.81 |
| 44N | 450 | 1.9 |
| 44C | 610 | 1.87 |
| 45N | 490 | 1.83 |
| 45C | 395 | 1.78 |
| 46N | 1145 | 1.81 |
| 46C | 1640 | 1.82 |
| 47N | 760 | 1.8 |
| 47C | 870 | 1.81 |
| 48N | 950 | 1.83 |
| 48C | 1270 | 1.8 |
| 49N | 657 | 1.79 |
| 49C | 760 | 1.78 |
| 50N | 800 | 1.88 |
| 50C | 940 | 1.86 |
| 51N | 370 | 1.9 |
| 51C | 497 | 1.92 |
| 52N | 778 | 1.89 |
| 52C | 980 | 1.83 |
| 53N | 500 | 1.76 |
| 53C | 510 | 1.78 |
| 54N | 419 | 1.66 |
| 54C | 426 | 1.65 |
| 55N | 1925 | 1.8 |
| 55C | 2650 | 1.81 |
| 56N | 1004 | 1.82 |
| 56C | 1450 | 1.83 |
| 57N | 560 | 1.9 |
| 57C | 780 | 1.89 |
| 58N | 760 | 1.8 |
| 58C | 790 | 1.84 |
| 59N | 1450 | 1.79 |
| 59C | 1780 | 1.81 |
| 60N | 580 | 1.77 |
| 60C | 379 | 1.54 |
| 61N | 553 | 1.67 |
| 61C | 828 | 1.72 |
| 62N | 611 | 1.85 |
| 62C | 789 | 1.82 |
| 63N | 856 | 1.8 |
| 63C | 927 | 1.81 |
| 64N | 667 | 1.83 |
| 64C | 789 | 1.82 |
| 65N | 1367 | 1.84 |
| 65C | 1870 | 1.79 |
| 66N | 1458 | 1.8 |
| 66C | 2230 | 1.81 |
| 67N | 567 | 1.78 |
| 67C | 870 | 1.79 |
| 68N | 500 | 1.82 |
| 68C | 659 | 1.82 |
| 69N | 943 | 1.78 |
| 69C | 1478 | 1.79 |
| 70N | 476 | 1.87 |
| 70C | 345 | 1.92 |
| 71N | 210 | 1.9 |
| 71C | 350 | 1.87 |
| 72N | 248 | 1.88 |
| 72C | 510 | 1.79 |
| 73N | 769 | 1.82 |
| 73C | 1277 | 1.8 |
| 74N | 187 | 1.67 |
| 74C | 489 | 1.75 |
| 75N | 567 | 1.76 |
| 75C | 932 | 1.78 |
| 76N | 520 | 1.79 |
| 76C | 450 | 1.78 |
| 77N | 1290 | 1.8 |
| 77C | 1467 | 1.81 |
| 78N | 760 | 1.82 |
| 78C | 770 | 1.78 |
| 79N | 891 | 1.79 |
| 79C | 756 | 1.77 |
| 80N | 672 | 1.78 |
| 80C | 770 | 1.83 |
| 81N | 1290 | 1.81 |
| 81C | 1678 | 1.79 |
| 82N | 2789 | 1.8 |
| 82C | 2981 | 1.8 |
| 83N | 901 | 1.81 |
| 83C | 1290 | 1.79 |
| 84N | 890 | 1.8 |
| 84C | 804 | 1.81 |
| 85N | 487 | 1.78 |
| 85C | 491 | 1.76 |
| 86N | 784 | 1.84 |
| 86C | 1299 | 1.81 |
| 87N | 1294 | 1.78 |
| 87C | 1967 | 1.79 |
| 88N | 560 | 1.76 |
| 88C | 873 | 1.78 |
| 89N | 960 | 1.81 |
| 89C | 1560 | 1.8 |
| 90N | 457 | 1.79 |
| 90C | 850 | 1.8 |
| 91N | 1370 | 1.81 |
| 91C | 1679 | 1.82 |
| 92N | 1320 | 1.81 |
| 92C | 1876 | 1.8 |
| 93N | 760 | 1.78 |
| 93C | 780 | 1.79 |
| 94N | 230 | 1.67 |
| 94C | 410 | 1.7 |
| 95N | 450 | 1.91 |
| 95C | 650 | 1.87 |
| 96N | 1120 | 1.81 |
| 96C | 1582 | 1.8 |
| 97N | 560 | 1.81 |
| 97C | 760 | 1.82 |
| 98N | 1154 | 1.79 |
| 98C | 1873 | 1.83 |
| 99N | 340 | 1.78 |
| 99C | 380 | 1.77 |
| 100N | 498 | 1.78 |
| 100C | 436 | 1.77 |
| 101N | 397 | 1.78 |
| 101C | 832 | 1.8 |
| 102N | 190 | 1.67 |
| 102C | 210 | 1.54 |
| 103N | 457 | 1.78 |
| 103C | 659 | 1.8 |
| 104N | 498 | 1.81 |
| 104C | 436 | 1.82 |
| 105N | 1170 | 1.8 |
| 105C | 1745 | 1.8 |
| 106N | 2130 | 1.81 |
| 106C | 2560 | 1.81 |
| 107N | 327 | 1.89 |
| 107C | 467 | 1.9 |
| 108N | 494 | 1.78 |
| 108C | 550 | 1.79 |
| 109N | 543 | 1.84 |
| 109C | 654 | 1.82 |
| 110N | 1280 | 1.8 |
| 110C | 1390 | 1.8 |
| 111N | 1987 | 1.81 |
| 111C | 2560 | 1.79 |
| 112N | 1942 | 1.8 |
| 112C | 2580 | 1.81 |
| 113N | 419 | 1.78 |
| 113C | 425 | 1.79 |
| 114N | 806 | 1.8 |
| 114C | 890 | 1.82 |
| 115N | 1524 | 1.82 |
| 115C | 1890 | 1.8 |
| 116N | 761 | 1.78 |
| 116C | 890 | 1.82 |
| 117N | 512 | 1.79 |
| 117C | 656 | 1.8 |
| 118N | 169 | 1.84 |
| 118C | 340 | 1.76 |
| 119N | 510 | 1.78 |
| 119C | 680 | 1.83 |
| 120N | 2178 | 1.8 |
| 120C | 2270 | 1.82 |
| 121N | 2370 | 1.81 |
| 121C | 2895 | 1.82 |
| 122N | 1990 | 1.8 |
| 122C | 2455 | 1.79 |
| 123N | 345 | 1.67 |
| 123C | 650 | 1.78 |
| 124N | 857 | 1.8 |
| 124C | 890 | 1.81 |
| 125N | 450 | 1.78 |
| 125C | 554 | 1.79 |
| 126N | 380 | 1.76 |
| 126C | 780 | 1.82 |
| 127N | 459 | 1.81 |
| 127C | 680 | 1.78 |
| 128N | 780 | 1.83 |
| 128C | 849 | 1.81 |
| 129N | 880 | 1.81 |
| 129C | 910 | 1.8 |
| 130N | 420 | 1.76 |
| 130C | 334 | 1.78 |
| 131N | 1189 | 1.83 |
| 131C | 1567 | 1.82 |
| 132N | 2190 | 1.81 |
| 132C | 2756 | 1.8 |
| 133N | 189 | 1.67 |
| 133C | 336 | 1.76 |
| 134N | 1789 | 1.83 |
| 134C | 2567 | 1.81 |
| 135N | 320 | 1.56 |
| 135C | 450 | 1.67 |
| 136N | 770 | 1.89 |
| 136C | 810 | 1.82 |
| 137N | 1245 | 1.8 |
| 137C | 1598 | 1.82 |
