# Supplementary material for: Aberrant Promoter Methylation of YAP Gene and its Subsequent Downregulation in Indian Breast Cancer Patients
Source: BMC Cancer. 2018 Jul 3;18:711. doi: 10.1186/s12885-018-4627-8 (PMC6031145; doi:10.1186/s12885-018-4627-8)
Supplement: Supplementary file 2 — Ct values of YAP and GAPDH mRNA. The Ct values for YAP and GAPDH mRNA for breast cancer cases and adjacent normal breast tissues used as control are shown in Additional file 2: Table S2. YAP mRNA expression was detected at the mRNA level in breast tumor and adjacent normal breast tissue using Real-time PCR. The expression was normalized against GAPDH expression. (DOCX 18 kb) [file 12885_2018_4627_MOESM2_ESM.docx]

| **Table S2: Ct values of YAP and GAPDH mRNA** | | | | |
| --- | --- | --- | --- | --- |
| **Samples** | **GAPDH Normal Ct value** | **YAP Normal Ct value** | **GAPDH Cancer Ct value** | **YAP Cancer Ct value** |
| 1 | 26.08 | 28.18 | 23.34 | 25.32 |
| 2 | 22.46 | 27.54 | 16.66 | 23.48 |
| 3 | 25.08 | 29.73 | 16.82 | 26.71 |
| 4 | 18.71 | 28 | 19.06 | 29.85 |
| 5 | 30.09 | 30.35 | 28.87 | 33.72 |
| 6 | 31.02 | 32.54 | 27.13 | 33.45 |
| 7 | 26.41 | 33.96 | 26.21 | 29.36 |
| 8 | 25.12 | 32.33 | 16.24 | 25.76 |
| 9 | 18.51 | 30.82 | 17.45 | 28.81 |
| 10 | 20.19 | 31.36 | 17.02 | 31.45 |
| 11 | 27.16 | 32.86 | 30.47 | 31.65 |
| 12 | 27.83 | 35.46 | 17.64 | 26.76 |
| 13 | 26.14 | 26.53 | 27.01 | 34.39 |
| 14 | 21.64 | 29.14 | 20.18 | 30.47 |
| 15 | 28.15 | 33.19 | 18.5 | 25.79 |
| 16 | 27.21 | 32.11 | 19 | 26.32 |
| 17 | 26.35 | 40.2 | 20.72 | 32.08 |
| 18 | 28 | 33 | 20.1 | 26.43 |
| 19 | 25.12 | 30.21 | 17.26 | 23.34 |
| 20 | 17.71 | 27 | 18.05 | 28.75 |
| 21 | 30.22 | 31.76 | 29.68 | 32.46 |
| 22 | 24.5 | 29.88 | 17.39 | 25.98 |
| 23 | 27.32 | 30.62 | 20.37 | 27.04 |
| 24 | 24.55 | 32.37 | 18.54 | 26.32 |
| 25 | 29.17 | 32.26 | 19.17 | 25 |
| 26 | 23 | 28.87 | 19.51 | 26.21 |
| 27 | 34.85 | 28.65 | 35.2 | 33.49 |
| 28 | 32.45 | 26.87 | 33.7 | 31.25 |
| 29 | 40.78 | 33.34 | 31.14 | 31.58 |
| 30 | 23.41 | 29.89 | 18.34 | 25.07 |
| 31 | 23.64 | 34 | 19.23 | 29 |
| 32 | 24.71 | 32.64 | 20.08 | 27.61 |
| 33 | 30.11 | 35.57 | 27.09 | 33.78 |
| 34 | 26.52 | 31.67 | 19.64 | 27.47 |
| 35 | 31.08 | 41.75 | 19.48 | 31.58 |
| 36 | 30.01 | 34.27 | 28.21 | 32.49 |
| 37 | 24.77 | 30.58 | 18.71 | 27.41 |
| 38 | 25.69 | 32 | 19.66 | 28.91 |
| 39 | 24.86 | 32.78 | 20.77 | 28.91 |
| 40 | 23.46 | 33.37 | 21.43 | 30.61 |
| 41 | 28.43 | 31.48 | 24.21 | 32.87 |
| 42 | 26.17 | 31.66 | 20.27 | 30.76 |
| 43 | 25.35 | 31.26 | 20.91 | 32.78 |
| 44 | 23.76 | 30.21 | 18.23 | 26.73 |
| 45 | 24.33 | 30.79 | 17.65 | 25.44 |
| 46 | 34.23 | 33.41 | 28.77 | 30.34 |
| 47 | 26.5 | 31.76 | 19 | 27.56 |
| 48 | 22.45 | 32.11 | 16.89 | 25.75 |
| 49 | 23.54 | 30.21 | 17.31 | 26.24 |
| 50 | 24.63 | 30.93 | 17.45 | 25.8 |
| 51 | 26.32 | 32.16 | 23.96 | 33.45 |
| 52 | 29.52 | 35.66 | 30.73 | 41.16 |
| 53 | 24.64 | 35.86 | 20.53 | 29.61 |
| 54 | 34.17 | 34.69 | 18.64 | 27.88 |
| 55 | 35.37 | 35.31 | 28.2 | 30.52 |
| 56 | 27.14 | 34.77 | 20.37 | 32.9 |
| 57 | 26.02 | 33.03 | 20.9 | 26.57 |
| 58 | 25.75 | 33.7 | 21.97 | 28.55 |
| 59 | 24.02 | 32.08 | 19.51 | 26.72 |
| 60 | 29.1 | 32.33 | 22.35 | 31.23 |
| 61 | 31.83 | 30.29 | 22.03 | 33.02 |
| 62 | 37.18 | 42.12 | 24.4 | 34.86 |
| 63 | 28.14 | 31.14 | 21.56 | 30.35 |
| 64 | 23.87 | 30.18 | 26.25 | 31.29 |
| 65 | 29.2 | 32.13 | 22.46 | 31.53 |
| 66 | 28.77 | 32.66 | 23.51 | 32.14 |
| 67 | 28 | 30.12 | 23.45 | 30.89 |
| 68 | 28.33 | 31.23 | 23.31 | 32.46 |
| 69 | 31.86 | 33.58 | 24.15 | 30.72 |
| 70 | 21.97 | 30.74 | 18.84 | 25.32 |
| 71 | 23.01 | 31.11 | 19.67 | 28.45 |
| 72 | 22.08 | 30.14 | 19.76 | 27.56 |
| 73 | 22.38 | 30.95 | 19.16 | 26.78 |
| 74 | 23.09 | 31.12 | 20.6 | 28.66 |
| 75 | 26.63 | 32.62 | 20.43 | 26 |
| 76 | 27.35 | 34.21 | 24.31 | 34.78 |
| 77 | 27.43 | 27.48 | 22.43 | 28.24 |
| 78 | 28.49 | 35.9 | 25.11 | 35.43 |
| 79 | 26.49 | 27 | 21.12 | 27.1 |
| 80 | 22.72 | 28.26 | 28.45 | 30.55 |
| 81 | 28.2 | 29.45 | 31.14 | 32.5 |
| 82 | 26.7 | 28.64 | 19.42 | 27.62 |
| 83 | 27.4 | 28.97 | 25.39 | 29.75 |
| 84 | 28.39 | 29.81 | 23.78 | 29.99 |
| 85 | 26.35 | 29.94 | 36.38 | 35.8 |
| 86 | 26.03 | 28.23 | 23.43 | 25.23 |
| 87 | 26.55 | 32.72 | 20.34 | 26.22 |
| 88 | 22.09 | 30.12 | 19.6 | 27.88 |
| 89 | 25.36 | 32.78 | 19.46 | 25.56 |
| 90 | 28.55 | 33.65 | 18.65 | 25.97 |
| 91 | 23.9 | 32.11 | 20.11 | 29.98 |
| 92 | 27.63 | 32.27 | 21.46 | 25.32 |
| 93 | 27.15 | 32.19 | 17.55 | 24.79 |
| 94 | 24 | 32.55 | 21.55 | 31.22 |
| 95 | 23.09 | 30.56 | 20.45 | 28.47 |
| 96 | 22.55 | 28.74 | 19.87 | 29.33 |
| 97 | 24.36 | 30.62 | 18.45 | 24.57 |
| 98 | 29.15 | 33.66 | 19.58 | 26.93 |
| 99 | 24.58 | 32.46 | 19.76 | 28.66 |
| 100 | 18.72 | 30.92 | 17.83 | 29.52 |
| 101 | 25.82 | 31.93 | 20.82 | 26.31 |
| 102 | 27.08 | 29.32 | 24.56 | 26.78 |
| 103 | 24.63 | 31.28 | 21.27 | 29.55 |
| 104 | 24.68 | 32.04 | 21.54 | 29.87 |
| 105 | 26.02 | 29.31 | 23.55 | 25.73 |
| 106 | 26.13 | 32.28 | 19.29 | 26.77 |
| 107 | 27.56 | 32.56 | 19.27 | 25.45 |
| 108 | 19.23 | 30.12 | 17.02 | 27.83 |
| 109 | 23.56 | 30.49 | 19.38 | 25.82 |
| 110 | 28.45 | 32.47 | 18.73 | 26.54 |
| 111 | 29.44 | 31.73 | 19.35 | 23.55 |
| 112 | 27.17 | 31.89 | 19.37 | 26.33 |
| 113 | 25.63 | 31.62 | 19.43 | 24.66 |
| 114 | 25.41 | 31.26 | 19.54 | 25.31 |
| 115 | 29.87 | 32.29 | 23.47 | 31.25 |
| 116 | 20.09 | 30.93 | 18.34 | 27.19 |
| 117 | 18.99 | 31.23 | 17.88 | 28.39 |
| 118 | 22.39 | 31.24 | 19.43 | 29.45 |
| 119 | 20.19 | 30.28 | 17.83 | 28.94 |
| 120 | 20.77 | 32.46 | 18.76 | 32.22 |
| 121 | 19.57 | 31.94 | 18.55 | 29.36 |
| 122 | 26.73 | 32.66 | 21.38 | 30.29 |
| 123 | 25.19 | 33.04 | 21.33 | 28.17 |
| 124 | 21.19 | 32.24 | 20.26 | 31.67 |
| 125 | 23.64 | 33.42 | 20.14 | 28.97 |
| 126 | 25.67 | 32.18 | 20.47 | 30.91 |
| 127 | 25.12 | 32.27 | 19.55 | 26.65 |
| 128 | 22.23 | 30.25 | 18.79 | 28.94 |
| 129 | 25.77 | 32.45 | 20.32 | 26.35 |
| 130 | 29.44 | 33.77 | 26.72 | 32.19 |
| 131 | 23.12 | 30.12 | 18.93 | 25.77 |
| 132 | 24.25 | 32.22 | 21.77 | 29.04 |
| 133 | 23.38 | 30.15 | 20.16 | 28.79 |
| 134 | 20.22 | 30.26 | 17.65 | 30.11 |
| 135 | 25.48 | 32.17 | 22.17 | 30.96 |
| 136 | 22.31 | 30 | 19.23 | 26.31 |
| 137 | 23.41 | 30.44 | 19.01 | 25.78 |
