# Supplementary figures and images for: Aberrant Promoter Methylation of YAP Gene and its Subsequent Downregulation in Indian Breast Cancer Patients
Source: BMC Cancer. 2018 Jul 3;18:711. doi: 10.1186/s12885-018-4627-8 (PMC6031145; doi:10.1186/s12885-018-4627-8)

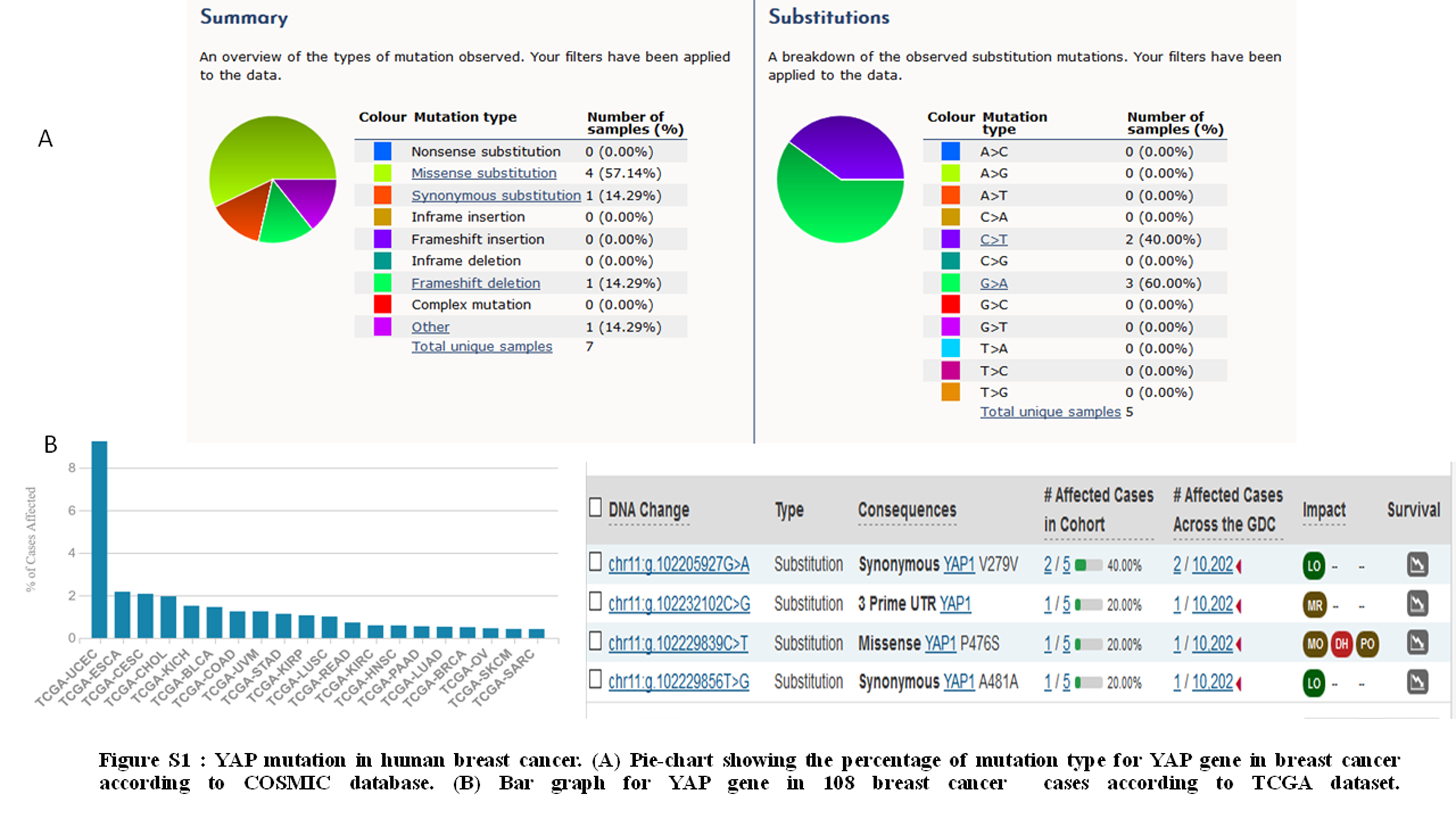

Supplement: Supplementary file 3 — YAP mutation in human breast cancer. TCGA and COSMIC database performed to analyse the mutations of YAP are shown in Additional file 3: Figure S1. The pie chart showing the information of mutations was generated using COSMIC databse. The data obtained using the cBioPortal for Cancer Genomics are shown in 108 cases affected by 102 mutations across 22 projects according to TCGA database. (TIF 4332 kb) [file 12885_2018_4627_MOESM3_ESM.tif]
